# Novel Polymeric Formulation for Removal of Gastrointestinal Polyps by Digestive Endoscopy

**DOI:** 10.3390/pharmaceutics12040322

**Published:** 2020-04-02

**Authors:** Cristina Moles-Aranda, Ana C. Calpena-Campmany, Lyda Halbaut-Bellowa, Victoria Díaz-Tomé, Francisco J. Otero-Espinar, José A. Morales-Molina, Beatriz Clares-Naveros

**Affiliations:** 1 Department of Pharmacy and Pharmaceutical Technology, Faculty of Pharmacy, University of Granada, University Campus of Cartuja, 18071 Granada, Spain; cmolesaranda@hotmail.com; 2 Department of Pharmacy and Pharmaceutical Technology and Physical Chemistry, Faculty of Pharmacy and Food Sciences, University of Barcelona, 27-31 Joan XXIII Ave., 08028 Barcelona, Spain; anacalpena@ub.edu (A.C.C.-C.); halbaut@ub.edu (L.H.-B.); 3 Department of Pharmacology, Pharmacy and Pharmaceutical Technology, Faculty of Pharmacy, Campus Vida, University of Santiago de Compostela, 15872 Santiago de Compostela, Spain; victoriadiaztome@gmail.com (V.D.-T.); francisco.otero@usc.es (F.J.O.-E.); 4 Pharmacy Department, Torrecárdenas Hospital, 04009 Almería, Spain

**Keywords:** endoscopic submucosal dissection (ESD), injectable polymeric solution, mucosal elevation, sodium carboxymethylcellulose, hyaluronic acid

## Abstract

Endoscopic mucosal resection (EMR) and endoscopic submucosal dissection (ESD) are two techniques used in the resection of gastrointestinal mucosal polyps. The aim of this work is the development and evaluation of an innovative polymeric solution containing sodium carboxymethylcellulose and hyaluronic acid. For this purpose, several mixtures of these two main components, as well as other components such as fructose, citric acid, and zinc, are evaluated in terms of physicochemical and microbiological properties, rheological behavior, extensibility, syringeability, and stability at different storage conditions. Furthermore, the potential production of mucosal elevation and duration is also studied by an ex vivo model using porcine stomach and colon. Results show that the developed polymeric solutions possess optimal values of pH, from 4.58 to 6.63, for their use in the gastrointestinal tract. The formulations exhibit both Newtonian and pseudoplastic behaviors with different viscosity values as a function of their composition. All formulations exhibit high stability properties and no bacterial or fungal growth is detected. MCS01 and MCS05 are the polymeric solutions with the best syringeability results. In this line, MCS05 is the formulation that provides the highest, 2.20 ± 0.18 cm and 1.40 ± 0.11 cm, and longest-lasting, for more than 120 min, elevation effect on porcine submucosal stomach and colon tissues, respectively. Thus, it can be concluded that polymeric solution MCS05 might be considered as a promising tool for use in human EMR and ESD.

## 1. Introduction

Endoscopic mucosal resection (EMR) and endoscopic submucosal dissection (ESD) are the two main techniques used for the removal of early-stage gastrointestinal (GI) neoplasms. Both are minimally invasive and efficient approaches for the treatment of these abnormal and excessive growths of tissue [1,2]. They enable the complete removal of suspect premalignant lesions with an efficacy greater than 90% [3]. Furthermore, they are particularly suitable for lesions smaller than 15–20 mm in diameter, which represent a low risk of metastasis. However, in those larger lesions, which are resected in a piecemeal fashion, ESD is proposed to be more useful but increases the risk of local recurrence [4]. The ESD technique allows an en bloc endoscopic resection of superficial lesions, providing an improved histopathological diagnosis and decreased local recurrence rate [5,6].

In both techniques, the creation of a submucosal tissue elevation is required. The elevation is achieved by submucosal injection solutions, leading to a submucosal fluid cushion. This procedure makes it possible to delimit the area to be resected (colorants, methylene blue, or carmine indigo), to separate the lesion from the muscularis propria layer and the complete resection of the lesion, avoiding the risk of perforation (bleeding) and injury to the GI wall. It also enables a faster patient recovery [7]. Hence, its use is essential to ensure the effectiveness and safety of the intervention, as well as the patient’s quality of life.

In Japan, 0.4% sodium hyaluronate submucosal injection solution is widely used in ESD, which has been reported to be useful and safe. Additionally, it is the only submucosal injection solution that has been approved in Japan [8].

In Europe, there is no commercially available submucosal injection for use in EMR and ESD. Several injectable solutions are used off-label, among them, saline solution is frequently used because of its low cost and ease of use. However, this type of solution does not provide sufficient mucosal elevation, and due to its poor viscosity, disseminates very fast in the mucosa, so it requires multiple injections. This may involve a high risk for perforation and bleeding, and thus a longer operative period, as well as a slower recovery and decreasing patient comfort [9]. In this context, some clinical guidelines, such as the European Society of Gastrointestinal Endoscopy (ESGE) guideline, suggests the use of submucosal injectates for EMR that are more viscous than normal saline [10].

Since there still is no clear consensus on what the solution of choice is, a wide range of submucosal injection solutions have been developed globally [11]. Solutions with dextrose and other more viscous and longer-lasting injection solutions including colloids (e.g., dextran), fibrinogen, and autologous blood [12], gelofusine [13], or sodium hyaluronate have been proposed [14]. Other solutions including ingredients such as hypromellose or glycerol have been also reported [14].

Nowadays, despite all these options, physicians remain without an effective solution that significantly contributes to the advancement of rapid and safe endoscopic treatments [11]. In fact, the main problems of endoscopists, when using these solutions, are the difficulty to prepare or administer them, the lack of consistency of the injected solution, and the short time duration of the lesion lift. Sometimes its high cost, absence of indication, and toxicity are problems that also arise [15].

Thus, taking into account the needs that the actual state of the art implies, the aim of this work is to develop novel polymeric mucoadhesive solutions for submucosal injection with clinical application in EMR and ESD to guarantee the efficiency of the treatment. The secondary aim is to evaluate the physicochemical properties, stability, and microbiological safety. Additionally, the outcome of the endoscopic treatment is evaluated in an ex vivo model.

## 2. Material and Methods

The excipients used in the elaboration of the mucosectomy solutions were all of pharmacopoeia grade. Sterile hyaluronic acid (HA) solution (Uromac^®^) was provided by Nakafarma S.L. (Oviedo, Spain). One percent methylene blue for injection was provided by a local Pharmacy (Granada, Spain), and 0.9% phosphate buffered solution (PBS) was supplied by Laboratorios Grifols S.A. (Barcelona, Spain). Highly purified sodium carboxymethylcellulose (Na-CMC), with viscosity 1500–4500 mPa·s, and citric acid were obtained from Guinama S.L.U. (Valencia, Spain). Meinsol Oligo-Zinc (1 mg Zn/mL) was obtained from Fresenius Kabi S.A.U. (Barcelona, Spain). All other chemicals were purchased from Sigma-Aldrich (Madrid, Spain) unless otherwise noted.

### 2.1. Formulation Design 

The compositions of developed polymeric solutions for submucosal injection are reported in Table 1. Briefly, the required amount of Na-CMC was accurately weighed and added to the right amount of PBS which was previously heated to 75 °C and continuously stirred at 800 rpm until total dissolution and obtaining a Na-CMC solution. Then, under these conditions, pre-weighed amounts of fructose and citric acid were added until complete dissolution. Subsequently, the required volumes of Zn solution and methylene blue were incorporated to the mixture. Finally, according to the type of solution, an adequate volume of HA solution was integrated. The final solution was then maintained under continuous stirring (1600 rpm) at room temperature for 5 h.

All solutions were elaborated under sterile conditions in Class ISO 5 horizontal laminar flow cabinets (former class 100). They were sterilized by means of mini-spike^®^ filters (B. Braun, Melsungen, Germany). The solutions were conditioned in BD Plastipak^®^Luer-lock^®^ syringes and stored at 8 °C and 25 °C (room temperature) for further analysis.

### 2.2. Physicochemical Characterization

#### 2.2.1. Appearance 

The physical appearance was studied by visual observation of samples stored at each temperature. Thus, parameters such as color or tendency to spontaneously form precipitates could be appreciated.

#### 2.2.2. pH Measurement 

The pH values’ determination of formulations was performed in triplicate using a digital pH/mV-meter micro-pH 200 (Crison Instruments S.A., Barcelona, Spain). Readings were recorded at two temperatures and pre-selected times for 6 months. Significant differences of pH over an adequate value in our gels could have indicated a degradation of the gel or a wrong elaboration.

#### 2.2.3. Rheological Behavior

Rheological characterization of formulations was performed at 8, 25, and 37 °C, using a rotational rheometer HAAKE Rheostress 1 (Thermo Fisher Scientific, Karlsruhe, Germany) equipped with cone and plate geometry (0.105 mm gap) with a fixed lower plate and a mobile upper cone Haake C60/2° Ti (60 mm diameter, 2° angle). The device was connected to a thermostatic circulator Thermo Haake Phoenix II + Haake C25P and a computer provided with the HAAKE RheoWin^®^ Job Manager v. 4.0 software (Thermo Electron Corporation, Karlsruhe, Germany) to carry out the test and HAAKE RheoWin^®^ Data Manager v. 4.0 software (Thermo Electron Corporation, Karlsruhe, Germany) to perform the analyses of the obtained data.

Viscosity and flow curves were recorded for 3 min during the ramp-up period from 0 to 100 s^−1^, 1 min at 100 s^−1^ (constant share rate period), and finally, 3 min during the ramp-down period from 100 to 0 s^−1^. Viscosity values of all samples were determined in triplicate, 24 h after preparation (*t*_0_) and at different times for 6 months (*t*_6_). Viscosity mean values (mPa·s) were determined at 100 s^−1^ from the constant share rate period of each viscosity curve. Readings of samples stored at 8 °C were performed at this temperature (just after removing them from the refrigerator) and at 37 °C (once they were warmed). Similarly, rheological readings of those samples stored at room temperature were also addressed at 25 and 37 °C.

Furthermore, data obtained from the flow curves were fitted to different mathematical equations to identify the model that provided the best overall match of the experimentally observed records:

τ = η × γ  Newton,(1)

τ = τ_0_ + η_p_ × γ  Bingham, (2)

τ = *k* × γ_n_  Ostwald-De-Waele, (3)

τ = τ_0_ + *k*_1_ × γ_n_ Herschel-Bulkley, (4)

τ^1/2^ = τ_0_^1/2^ + *k*_1_ × γ^1/2^ Casson, (5)

τ = γ × (η_∞_ + (η_0_ − η_∞_)/(1 + (γ/γ_0_)*^n^*) Cross, (6)

where τ is the shear stress, γ is the shear rate (1/s), τ_0_ is the yield shear stress (Pa), η_p_ is the constant plastic viscosity (Pa·s), η_0_ is the zero shear viscosity (Pa·s), *k* is the consistency (s) and *n* is the flow index, the different values of *n* indicate the fluid behavior. For a Newtonian fluid, *n* = 1. If *n* < 1, the fluid is called pseudoplastic; if *n* > 1, the fluid is dilatant. The adequacy of the rheological profiles to each mathematical model was based on the highest correlation coefficient value (r) and the lowest chi-square value.

#### 2.2.4. Extensibility 

The extensibility test was performed on the basis of the method previously described by Sanz et al. [16]. An amount of 0.03 g of sample was placed between 2 glass slides of 20 cm^2^, as centered as possible. Force was generated onto the upper plate by adding known weights (200, 300, and 400 g), so the sample was compressed to uniform thickness. After 60 s, the weights were removed, and the area of the sample was measured. Samples were tested in triplicate for each weight at 37 °C. Furthermore, experimental results were fitted to mathematical models in an attempt to predict its behavior and compare formulations among them. For this task the Prism^®^ v. 5.0 software (GraphPad Software, Inc., San Diego, CA, USA) was used.

#### 2.2.5. Syringeability

This test was conducted using a Shimadzu AGS-X series (Shimadzu Europa GmbH, Duisburg, Germany) universal test machine with a 1000 N load cell. Syringes of 1, 5, and 50 mL (23G needles) were assembled on a special support that kept them fixed vertically. They were loaded with the formulations (1, 5, or 50 mL, as appropriate) and, consecutively, the plunger was attached to the upper support, which is connected to the load cell.

The methodology consisted of a simple compression method in which the crossbar descends at a constant speed of 2 mm/s, moving 20 mm. During this time, the force based on displacement was recorded in triplicate. The work was calculated from the area under the resultant force-displacement curve.

### 2.3. Stability of Polymeric Solutions

Samples were stored at 8 and 25 °C (room temperature) for 6 months. Different measures were performed during this period. Analyses comprised the evaluation of quantifiable parameters which could vary during storage, such as appearance, pH, rheological behavior, and microbial growth.

On the other hand, in order to predict the long-term stability of formulations, measurements of samples (*n* = 3) were accomplished by multiple light scattering at room temperature using the Turbiscan^®^ Lab (Formulaction Co., L’Union, France). The light source is a pulsed near infrared (λ = 880 nm). Undiluted samples were placed and kept on cylindrical glass measuring cells which were completely scanned by a reading head, obtaining a pattern of the light flow as a function of the sample height. This pattern relates to a macroscopic fingerprint obtained through the data from transmission light intensity and the data from reflection (backscattering).

### 2.4. Microbiological Analysis 

Polymeric solutions were cultured at 0, 7, 15, and 30 days after elaboration. Microbiological sterility of all the samples was determined in triplicate at each time point. Samples were inoculated in culture media BacT/ALERT^®^ FA plus (bioMérieux, Marcy l’Etoile, France). After 24–48 h, it was detected if a microbe grew, and after 5 days, the validation of the analysis was obtained. To evaluate the bacterial contamination, plates of Columbia agar with 5% sheep blood (COS) and plates of chocolate agar PolyViteX^®^ (bioMérieux, Marcy l’Etoile, France) were used. Both were incubated in CO_2_ atmosphere for 5 days at 37 °C. Then, to test any fungal contamination, samples were spread on a plate with Sabouraud gentamicin chloramphenicol 2 agar (bioMérieux, Marcy l’Etoile, France) medium. In this case, plates were incubated under aerobic conditions at 37 °C for 5 days.

### 2.5. Ex Vivo Submucosal Elevation Studies

The ex vivo submucosal elevation study was performed on the basis of the method previously described by Uraoka et al. [7], using fresh resected porcine stomach and colon specimens. Porcine organs were obtained from the Animal Facility at the Bellvitge Campus of Barcelona University (Barcelona, Spain). The animals, male pigs, weighing 30–40 kg, were sacrificed for other purposes through an overdose of sodium pentobarbital anesthesia. Fresh stomach and colon were obtained immediately after sacrifice and they were placed in Hanks balanced salt solution and refrigerated until the beginning of experiments (not more than 24 h). 

Prior to injection, tissue specimens were cut into 5 × 5 cm pieces and were fixed flat on a stainless steel plate without tension, which was then mounted on a thermostatic bath water at 37 °C. Then, according to preliminary experiments aimed at the optimization of the settings and intensive literature research an injection volume of 10 mL was selected as the optimal volume for our study. On this basis, 10 mL of each polymeric solution were injected tangentially into the submucosa of the specimens through the mucosal surface using a 23-gauge needle by an investigator other than the investigator who addressed further measurements. Submucosal elevation heights were observed from the lateral position and recorded directly by the investigator using a transparent graduated scale [17] immediately after the injection at pre-established time intervals, 1, 3, 5, 7, 10, 15, 30, 60, 90, and 120 min after the injection. The study was performed at 37 °C to mimic physiological conditions. Three independent measurements were performed for each sample (graphic abstract). Furthermore, experimental results were fitted to mathematical models in an attempt to predict its behavior and compare formulations among them. For this task the Prism^®^ v. 5.0 software (GraphPad Software, Inc., San Diego, CA, USA) was used.

### 2.6. Statistical Analysis

Data were statistically analyzed by one-way ANOVA, followed by the Student’s *t*-test and represented as the mean of *n* replicates ± SD. In the case of the syringeability results, Tukey’s multiple comparison test was performed. The level of significance was set at *p* < 0.05 using Prism^®^, v. 5.0 software (GraphPad Software, Inc., San Diego, CA, USA).

## 3. Results

### 3.1. Physicochemical Characterization

After preparation (*t*_0_), all polymeric solutions were transparent and slightly blue, due to the methylene blue due. No signs of precipitation or other alteration phenomena were observed. The appearance remained unaltered at 8 and 25 °C for 6 months.

In Table 2 are reported the obtained results of pH measurements 24 h after elaboration and after 6 months stored at 8 °C and room temperature. It could be observed that pH values of polymeric solutions at 24 h (*t*_0_) ranged from 4.58 to 6.63, and between 4.46 and 6.18 after the 6 months storage period (*t*_6_). No significant differences in pH values were observed between similar samples after storage at both temperatures.

Table 3 displays the results of the rheological characterization of the developed polymeric solutions at 24 h (*t*_0_), measured at 8, 25, and 37 °C. As can be observed from formulations, MSC01 and MSC05 exhibited a Newtonian behavior. MSC03 showed pseudoplastic behavior, and MSC04 displayed both patterns, pseudoplastic and Newtonian, in samples assayed at 8 and 37 °C, respectively. Viscosity values were higher for the polymeric solutions MSC03 and MSC04.

On the other hand, Table 4 displays the results of the rheological characterization of developed polymeric solutions after 6 months (*t*_6_), measured at 8, 25, and 37 °C. Regarding the influence of the storage period, no significant changes were observed in the rheological properties.

Regarding the influence of the conservation time, no significant changes were observed for all the analyzed solutions.

**Figure 1:**
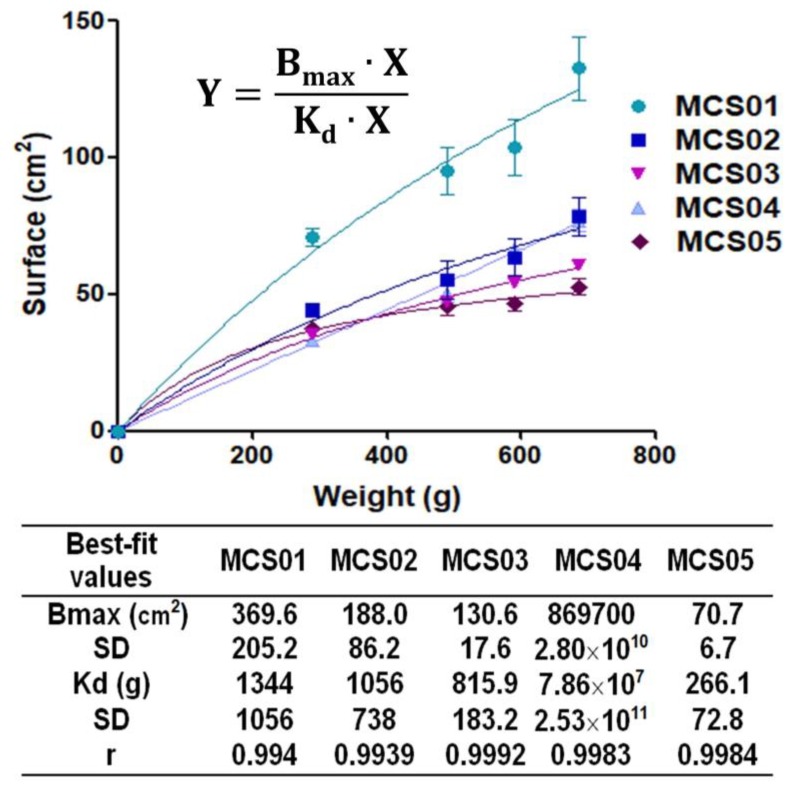
Extensibility results of polymeric solutions (adjusted to hyperbola equation). Data are expressed as mean ± SD (*n* = 3). The model fitting of experimental data to the hyperbola equation is also depicted.

Extensibility results are depicted in Figure 1. It can be observed that the statistically significant highest extensibility value was exhibited by MCS01 polymeric solution followed by MCS02. The formulations with the lowest extensibility were MSC03 and MSC05. The hyperbola equation was the model with the best adjustment quality (see graphs and model parameters in Figure 1).

**Figure 2:**
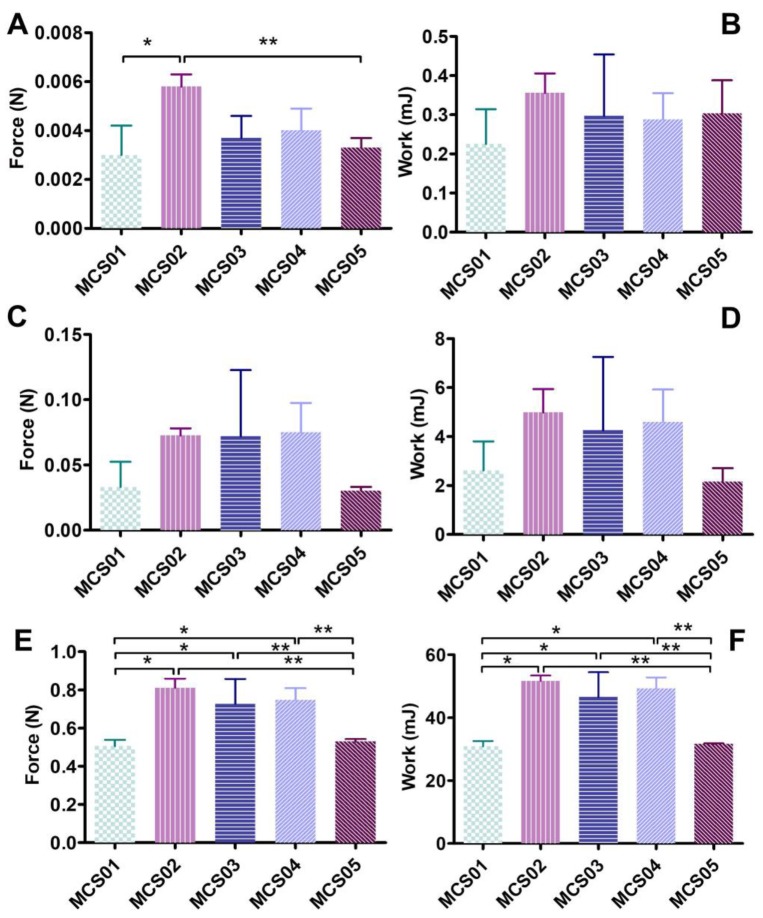
Syringeability results of maximum force and work, respectively; (**A**,**B**) using 1 mL syringes; (**C**,**D**) using 5 mL syringes; (**E**,**F**) using 50 mL syringes. Data are expressed as mean ± SD (*n* = 3). * Statistically significant differences regarding MCS01 (*p* < 0.05). ** Statistically significant differences regarding MCS05 (*p* < 0.05).

Figure 2 shows the syringeability results of maximum force and work for the developed polymeric solutions packaged in 1, 5, and 50 mL syringes. Twenty-three G needles were used, since these are the most common gauge utilized in the clinical practice [7]. It can be clearly observed that the polymeric solutions MCS01 and MCS05 are the formulations for which the statistical lowest force needs to be applied, and thus are those with the best syringeability properties in the three volume syringes assayed. No significant differences were shown among MCS02, MCS03, and MCS04. Concretely, the force used in the case of MCS01 and MCS05 were 0.0033 N in both cases in 1 mL syringes, 0.0330 and 0.0302 N, respectively, in 5 mL syringes, and 0.5064 and 0.5293, respectively, in 50 mL syringes. No statistical differences were observed between MCS01 and MCS05.

### 3.2. Optical and Microbiological Stability

**Figure 3:**
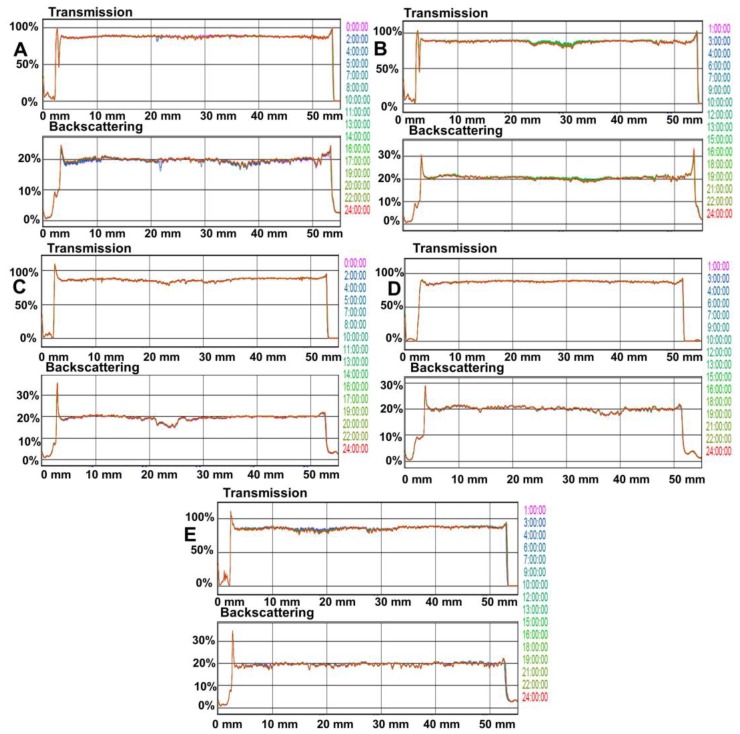
Transmission and backscattering profiles of polymeric solutions after 24 h (*t*_0_); (**A**) MCS01; (**B**) MCS02; (**C**) MCS03; (**D**) MCS04; (**E**) MCS05.

Figure 3 shows the transmission and backscattering profiles of polymeric solutions 24 h after preparation (*t*_0_). The left side of the graphics corresponds to the bottom of the vial, whereas the right side corresponds to the top. If a sedimentation process is produced, a backscattering increase versus time at the bottom of the vial is observed. By contrast, when the creaming process is produced, an increase of backscattering versus time on the top of the vial is observed. If the destabilization phenomenon occurs due to aggregation, a backscattering increase versus time can be observed over the whole height of the vial. Variations above ±10% mean instable formulations.

The superposition of the transmission and/or reflection signals from 0 to 24 h shows the formulation stability, indicating the absence of any destabilization processes. This behavior is the same for all polymeric solutions. Furthermore, the analysis during storage did not show any sign of destabilization processes independently of the time of analysis or the storage conditions. In conclusion, the polymeric solutions constitute homogeneous dispersions.

On the other hand, no bacterial or fungal growth in any of the samples was observed, which confirmed the microbiological stability and safety of the formulations for at least 30 days.

### 3.3. Ex Vivo Submucosal Elevation Study 

**Figure 4:**
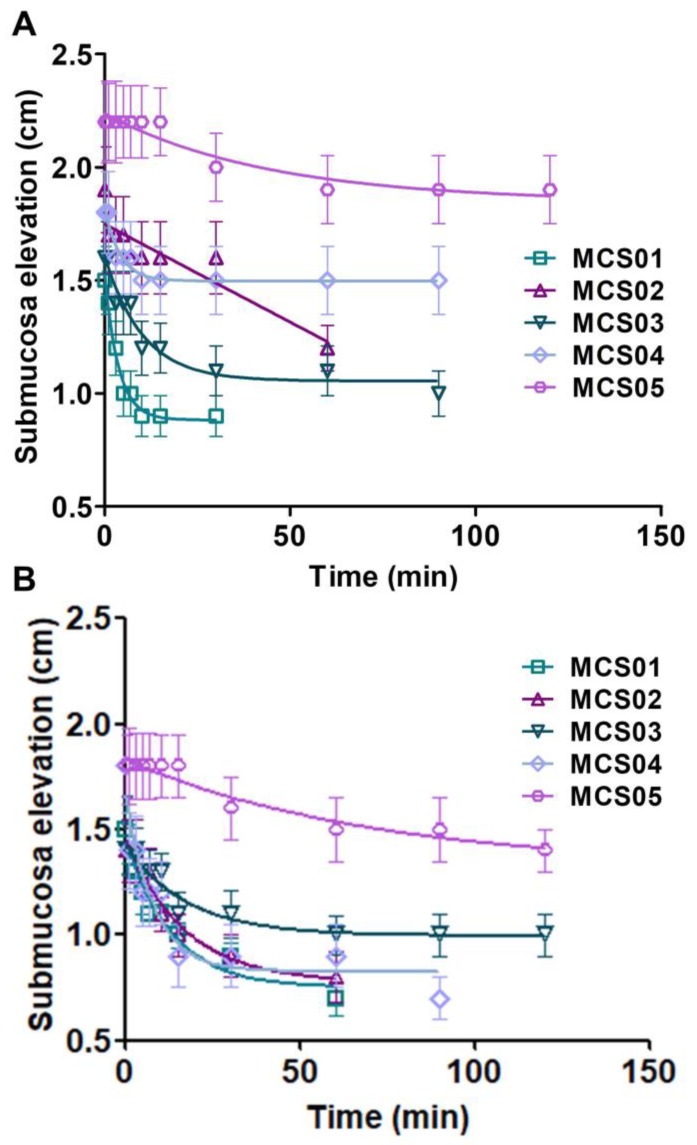
Submucosal elevation vs. time of polymeric solutions; (**A**) in stomach tissue; (**B**) in colon tissue. Data are expressed as mean ± SD of at least three independent experiments.

Figure 4 shows submucosal elevations (cm) of each formulation injected into stomach submucosa (Figure 4A) and colon submucosa (Figure 4B) as a function of time. It could be observed that the polymeric solution MCS05 was the formulation which statistically provided more elevation, reaching 2.20 ± 0.18 cm and 1.40 ± 0.11 cm for the stomach and colon, respectively, and a lasting effect, for more than 120 min, in both types of tissues followed by MCS04 in stomach submucosa and MCS03 in colon submucosa.

A correlation test between the maximum elevation time and extensibility in terms of area under the curve (AUC) was conducted. The best fitting was obtained with a polynomial second order model for both the stomach and colon. The model equation with correlation coefficients (*r*) of 0.946 and 0.981 for the stomach and colon, respectively, is the following:

*Y* = *a* + *b* × *X* + *c* × *X*^2^ Polynomial second order, (7)

In the case of the stomach, obtained data were *a* = 491.3 ± 140.0, *b* = −0.0228 ± 0.0085, and *c* = 2.734 × 10^−7^ ± 1.169 × 10^−7^. On the other hand, colon data were *a* = 649.5 ± 73.50, *b* = −0.0324 ± 0.0045, and *c* = 4.173 × 10^−7^ ± 6.134 × 10^−7^.

**Figure 5:**
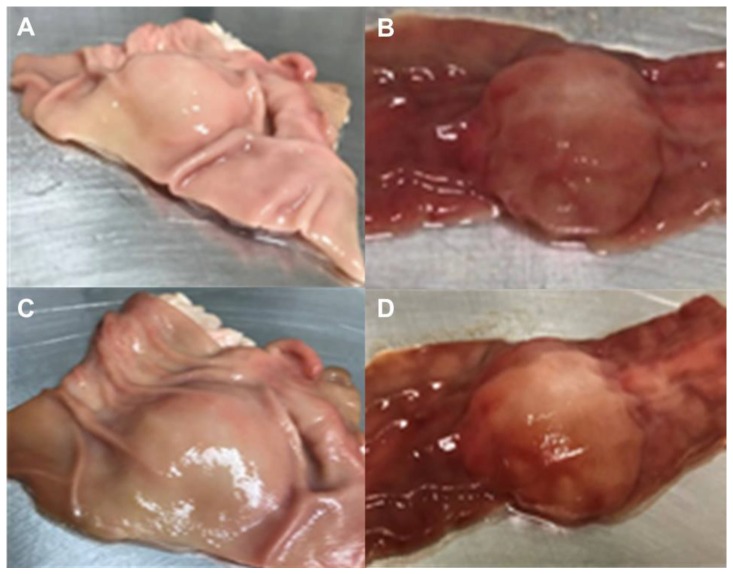
Submucosal elevation; (**A**) porcine stomach just after injection; (**B**) porcine colon just after injection; (**C**) porcine stomach 90 min after injection; (**D**) porcine colon 90 min after injection.

In Figure 5 is shown the changes in submucosal elevation immediately and 90 min after the injection of polymeric solution MCS05.

## 4. Discussion

The purpose of this study was the development and evaluation of the performance characteristics of a submucosal injection for ESD, which included as the main components HA, Na-CMC, fructose, and other ingredients. The research of formulations with specific properties appropriate for ESD is nowadays an important challenge. This formulation should meet some needs to be clinically applicable. This formulation should be biocompatible, easily injectable, provide a long-lasting and sufficiently high submucosal cushion of the lesion, allow the visualization of the edges of the lesion, prolonged physicochemical and microbiological stability, as well as low cost.

On the basis of these requirements, biocompatible ingredients were selected for the present research. HA is a glycosaminoglycan which is naturally found in connective tissue. The use of HA in mucosal injections for EMR and ESD has been reported even under in vivo conditions with living animal models, obtaining optimal results [18]. Furthermore, Na-CMC is cellulose ether with a carboxymethyl radical introduced into the hydroxyl group, which is also used as a thickening agent, binder, film former, and hydrophilic matrix material of pharmaceutical products. It has optimal biocompatibility and chemical stability. It has been also assayed for ESD in animal models with good results [19]. Fructose is utilized as a non-toxic hypertonic agent and it is able to increase the viscoelasticity of HA by the cross-linking of HA molecules [20]. Citric acid was added to stabilize pH, because pre-formulation studies showed that variations of pH resulted in precipitation residues. Moreover, the inclusion of Zn in polymeric solutions is motivated by the important role of this element for intestinal homeostasis, since several antioxidant enzymes are Zn-dependent, and Zn metabolism could be altered during inflammatory processes. Equally, Zn is related to the gene expression of inflammatory cytokines also involved in intestinal wound healing and epithelial repair [21]. Zn also promotes the stabilization of chains of HA [22,23]. Finally, the inclusion of methylene blue as a staining dye was aimed at identifying the lateral and deep margins of the target lesion.

It is particularly striking that an extensive bibliography on the development of formulations for EDS and EMR does not consider the importance of pH of submucosal injection formulation. Values of pH not only could have a significant impact on the rheological stability of the solution, but also could modify the biological pH of the GI tract. For these reasons, values of pH between 4 and 6 are recommended to be biocompatible values. Our results of pH showed these biocompatible values between 4 and 6. Additionally, no variations were recorded after the storage period, which was indicative of its stability and safety.

Rheological properties of submucosal injectable formulations EDS and EMR is certainly an important issue to be investigated. A very viscous formulation would hinder the injection process. Surely, the administration of a too viscous formulation would need a submucosal injection needle catheter to minimize injection resistance which could lead to submucosal perforation and bleeding. Contrary, a diluted formulation excessively diffuses quickly when injected into the submucosal layer and might be unable to dissect the mucosal layer from the muscular layer. Besides, the mucosal elevation would disappear quickly, due to its rapid removal from the lesion. Consequently, repeated injections are necessary for prolonging the procedure time [24]. In fact, the ESGE guidelines have recommended more viscous injection solutions over the use of normal saline for endoscopic mucosal resections [10]. In our study, rotational rheology serves to characterize the flow behavior of the final system, which provides important information about the functional properties of the final product during the administration (mechanical behavior), as well as a quality control parameter in basic operations such as pumping, mixing, packaging, storage, and physical stability. As can be observed in Table 3, polymeric solutions exhibited both Newtonian and pseudoplastic behaviors with a wide rank of viscosity values (1.10 ± 0.03–37.98 ± 0.05 mPa·s). In the first case, for MCS01, MCS04 in samples assayed at 37 °C, and MCS05, a constant viscosity regardless of the shear rate was found. In contrast, rheological profiles of MCS02, MCS03, and MCS04 in samples assayed at 8 °C showed shear-rate-dependent viscosity. All those showing pseudoplastic behavior fit to the Ostwald de Waele mathematical model (Equation (3)). This model is commonly used to describe the viscosity–shear rate relationship of non-Newtonian fluids, where the viscosity is described as a product of the flow consistency index (k) and the shear rate (γ) to the power of n (power law index; dimensionless). Thus, as the viscosity of a non-Newtonian fluid is shear-rate-dependent, it could change over the needle cross-section in response to the varying shear rate during the administration process [25]. In the light of this, and given that the viscosity of MCS05 ranged in intermediate values, this polymeric solution set out on the path to becoming the most promising. Rheological stability was confirmed by obtained results after a 6-month storage period (Table 4). Although previous studies by different authors [24,25,26,27] have concluded that pseudoplastic fluids can be more effective than Newtonian fluids, in our investigation, the evidence indicates that the MCS05 formulation with Newtonian behavior was more promising that other pseudoplastic formulations (MCS02, MCS03, and MCS04). In fact, other studies have reported that more viscous solutions caused the dissection of the mucosal layer from the muscular layer, due to viscous solutions not diffusing after injection into the submucosal layer, thus more mechanical expanding pressure had to be applied [28].

The other thinning systems under study were considerably more viscous than MCS05, however, they required greater force and work for the injection. Moreover, they resulted in being more sensitive to changes in temperature, which represents some additional inconveniences.

Extensibility results showed that MCS01 polymeric solution statistically possessed the highest value. This might be due to the absence of Na-CMC. On the contrary, the rest of the polymeric solutions exhibited similar patterns (Figure 1). Na-CMC confers viscosity in low concentration, improving the administration properties of formulations.

The syringeability study determines the maximum force and the work needed to expel the formulations from the syringe. In the case of 1 mL syringes, a limit of 0.38 J was established as the best value for parenteral administration formulations [16,29]. Values obtained with a 1 mL syringe are close to or below this value, so they should be good for administration. When results of studies carried out with greater volume syringes were compared, allowing to establish differences between formulations, it was observed that MCS01 and MCS05 were the ones with the best syringeability properties (Figure 2). This result outcome strengthened the MCS05 polymeric solution as the best formulation at this stage of the research.

The stability of all polymeric solutions was demonstrated by the optical study. In essence, this technique detects size or location changes in the solutions. Therefore, it is considered as a technique that predicts long-term stability, being able to detect the formula destabilization earlier than the classical stability methods. Results showed no variation in light patterns. Concretely, MCS05 showed a continuous line along the graph (Figure 3E) for the transmission and backscattering signal, which lets us demonstrate that this polymeric solution constitutes homogeneous dispersion with high stability and thus assures the safety of the potential clinical treatment.

To improve the efficacy and safety of endoscopic submucosal dissection techniques, the quality and duration of the submucosal cushion must be assayed because they are key parameters. For these purposes, ex vivo models using porcine stomach and colon is a well-accepted technique usually used to test the performances of formulations [26]. Through this model, the in vivo conditions of the human GI tract can be reproduced, and a more accurate measure of the elevation can be addressed. The polymeric solutions performances for cushion development confirmed that MCS05 was the most appropriate polymeric solution for injection, which provided the most lasting effect and the highest submucosal elevation. This elevation would provide a gap between the mucosal and deeper layer of tissues, which facilitates the clinical resection of lesions.

## 5. Conclusions

In this research, a high performance polymeric solution was effectively developed. This polymeric solution containing Na-CMC, HA, fructose, citric acid, Zn, methylene blue, and PBS showed optimal physicochemical properties for its use as an injectable, as well as being of a Newtonian nature, with an appropriate viscosity value to facilitate its administration and providing a long lasting effect and tissue elevation. The safety of the treatment was assured by its physicochemical and microbiological stability. Therefore, such important results open the door to considering this polymeric solution as a suitable candidate for future in vivo studies and make this MCS05 formulation a promising tool for the clinical treatment of EDS.

## 6. Patents

J.A.M.-M. and B.C.-N. are co-inventors on a provisional patent application encompassing the technology described in this manuscript.

## Figures and Tables

**Table 1:** Compositions (% *w*/*v*) of polymeric solution formulations.

Ingredients	MCS01	MCS02	MCS03	MCS04	MCS05
Na-CMC (% *w*/*v*)	-	0.2	0.2	0.1	0.1
HA (% *v*/*v*)	0.03	-	0.03	0.12	0.12
Fructose (% *w*/*v*)	17	17	17	17	17
Citric acid (% *v*/*v*)	-	-	-	-	0.02
Zinc (% *w*/*v*)	-	-	-	-	0.02
Methylene blue (% *v*/*v*)	0.0005	0.0005	0.0005	0.0005	0.0005
PBS (mL)	q.s. 100	q.s. 100	q.s. 100	q.s. 100	q.s. 100

**Table 2:** pH values in samples stored at 8 and 25 °C, 24 h after elaboration (*t*_0_) and after 6 months (*t*_6_). Values represent mean ± SD (*n* = 3).

Formulation	pH (*t*_0_)		pH (*t*_6_)	
	8 °C	25 °C	8 °C	25 °C
MCS01	5.50 ± 0.01	5.63 ± 0.01	5.49 ± 0.02	5.42 ± 0.08
MCS02	5.88 ± 0.05	5.56 ± 0.02	5.77 ± 0.05	5.38 ± 0.05
MCS03	5.82 ± 0.09	5.75 ± 0.01	5.29 ± 0.07	5.89 ± 0.02
MCS04	6.19 ± 0.03	6.23 ± 0.02	6.07 ± 0.05	6.18 ± 0.04
MCS05	4.63 ± 0.02	4.58 ± 0.03	4.61 ± 0.01	4.46 ± 0.02

**Table 3:** Rheological characterization 24 h after preparation (*t*_0_) of polymeric solutions at different temperatures of measurement and storage.

Formulation	Measurement Temperature (°C)	Storage Temperature (°C)	Mathematical Model Fitting		Rheological Behavior	Viscosity (mPa·s)
			Ramp-Up Stretch	Ramp-Down Stretch		
**MCS01**	8	8	OdW ^1^ *r* = 0.9996	OdW *r* = 0.9998	Newtonian	2.23 ± 0.04
		25	Newton *r* = 0.9978	Newton *r* = 0.9975	Newtonian	1.48 ± 0.03
	37	8	Newton *r* = 0.9995	Newton *r* = 0.9957	Newtonian	1.16 ± 0.03
		25	Newton *r* = 0.9995	Newton *r* = 0.9941	Newtonian	1.10 ± 0.03
**MCS02**	8	8	OdW *r* = 0.9999	OdW *r* = 0.9998	Pseudoplastic	25.35 ± 0.05
		25	OdW *r* = 0.9999	OdW *r* = 0.9998	Pseudoplastic	5.35 ± 0.03
	37	8	OdW *r* = 0.9999	OdW *r* = 0.9998	Pseudoplastic	13.14 ± 0.05
		25	OdW *r* = 0.9994	OdW *r* = 0.9995	Pseudoplastic	3.25 ± 0.04
**MCS03**	8	8	OdW *r* = 0.9999	OdW *r* = 0.9998	Pseudoplastic	21.27 ± 0.08
		25	OdW *r* = 0.9999	OdW *r* = 0.9998	Pseudoplastic	13.25 ± 0.05
	37	8	OdW *r* = 0.9999	OdW *r* = 0.9999	Pseudoplastic	13.62 ± 0.04
		25	OdW *r* = 0.9999	OdW *r* = 0.9998	Pseudoplastic	13.70 ± 0.04
**MCS04**	8	8	OdW *r* = 0.9996	OdW *r* = 0.9997	Pseudoplastic	37.98 ± 0.05
		25	OdW *r* = 0.9999	OdW *r* = 0.9998	Pseudoplastic	21.94 ± 0.01
	37	8	Newton *r* = 0.995	Newton *r* = 0.9925	Newtonian	17.28 ± 0.03
		25	Newton *r* = 0.9934	Newton *r* = 0.9955	Newtonian	17.32 ± 0.03
**MCS05**	8	8	Newton *r* = 0.9983	Newton *r* = 0.9993	Newtonian	8.47 ± 0.01
		25	Newton *r* = 0.9995	Newton *r* = 0.9997	Newtonian	4.42 ± 0.01
	37	8	Newton *r* = 0.9999	Newton *r* = 0.9999	Newtonian	3.50 ± 0.02
		25	Newton *r* = 0.9997	Newton *r* = 0.9989	Newtonian	3.47 ± 0.03

^1^ OdW = Ostwald de Waele.

**Table 4:** Rheological characterization 6 months after preparation (*t*_6_) of polymeric solutions at different temperatures of measurement and storage.

Formulation	Measurement Temperature (°C)	Storage Temperature (°C)	Mathematical Model Fitting		Rheological Behavior	Viscosity (mPa·s)
			Ramp-Up Stretch	Ramp-Down Stretch		
**MCS01**	8	8	OdW ^1^ *r* = 0.9998	OdW *r* = 0.9998	Newtonian	2.28 ± 0.04
		25	Newton *r* = 0.9945	Newton *r* = 0.9925	Newtonian	1.48 ± 0.04
	37	8	Newton *r* = 0.9907	Newton *r* = 0.9888	Newtonian	1.14 ± 0.04
		25	Newton *r* = 0.9781	Newton *r* = 0.9889	Newtonian	1.13 ± 0.04
**MCS02**	8	8	OdW *r* = 0.9998	OdW *r* = 0.9999	Pseudoplastic	26.88 ± 0.06
		25	OdW *r* = 0.9997	OdW *r* = 1.0000	Pseudoplastic	4.38 ± 0.05
	37	8	OdW *r* = 0.9999	OdW *r* = 0.9998	Pseudoplastic	13.07 ± 0.05
		25	OdW *r* = 0.9962	OdW *r* = 0.9961	Pseudoplastic	3.80 ± 0.05
**MCS03**	8	8	OdW *r* = 0.9998	OdW *r* = 0.9999	Pseudoplastic	23.61 ± 0.03
		25	OdW *r* = 0.9997	OdW *r* = 1.0000	Pseudoplastic	12.28 ± 0.02
	37	8	OdW *r* = 0.9999	OdW *r* = 0.9998	Pseudoplastic	12.92 ± 0.05
		25	OdW *r* = 0.9998	OdW *r* = 0.9999	Pseudoplastic	12.03 ± 0.05
**MCS04**	8	8	OdW *r* = 0.9998	OdW *r* = 0.9996	Pseudoplastic	38.53 ± 0.05
		25	OdW *r* = 0.9997	OdW *r* = 0.9999	Pseudoplastic	22.46 ± 0.09
	37	8	Newton *r* = 0.9988	Newton *r* = 0.9996	Newtonian	16.74 ± 0.06
		25	Newton *r* = 0.9934	Newton *r* = 0.9955	Newtonian	17.11 ± 0.03
**MCS05**	8	8	Newton *r* = 0.9935	Newton *r* = 0.9990	Newtonian	8.55 ± 0.03
		25	Newton *r* = 0.9988	Newton *r* = 0.9996	Newtonian	4.40 ± 0.02
	37	8	Newton *r* = 0.9990	Newton *r* = 0.9989	Newtonian	3.25 ± 0.04
		25	Newton *r* = 0.9998	Newton *r* = 0.997	Newtonian	3.50 ± 0.01

^1^ OdW = Ostwald de Waele.
